# Rapid expansion and international spread of M1_UK_ in the post-pandemic UK upsurge of *Streptococcus pyogenes*

**DOI:** 10.1038/s41467-024-47929-7

**Published:** 2024-05-10

**Authors:** Ana Vieira, Yu Wan, Yan Ryan, Ho Kwong Li, Rebecca L. Guy, Maria Papangeli, Kristin K. Huse, Lucy C. Reeves, Valerie W. C. Soo, Roger Daniel, Alessandra Harley, Karen Broughton, Chenchal Dhami, Mark Ganner, Marjorie A. Ganner, Zaynab Mumin, Maryam Razaei, Emma Rundberg, Rufat Mammadov, Ewurabena A. Mills, Vincenzo Sgro, Kai Yi Mok, Xavier Didelot, Nicholas J. Croucher, Elita Jauneikaite, Theresa Lamagni, Colin S. Brown, Juliana Coelho, Shiranee Sriskandan

**Affiliations:** 1 Department of Infectious Disease, Imperial College London, London, UK; 2 Centre for Bacterial Resistance Biology, Imperial College London, London, UK; 3 NIHR Health Protection Research Unit in Healthcare-associated Infections and AMR, Imperial College London, London, UK; 4 Healthcare-Associated Infections, Fungal, AMR, AMU, and Sepsis Division, UK Health Security Agency, London, UK; 5 Reference Services Division, UK Health Security Agency, London, UK; 6 School of Life Sciences and Department of Statistics, University of Warwick, Coventry, UK; 7 School of Public Health, Imperial College London, London, UK; 8 MRC Centre for Global Infectious Disease Analysis, Imperial College London, London, UK

**Keywords:** Bacterial genetics, Bacterial infection

## Abstract

The UK observed a marked increase in scarlet fever and invasive group A streptococcal infection in 2022 with severe outcomes in children and similar trends worldwide. Here we report lineage M1_UK_ to be the dominant source of invasive infections in this upsurge. Compared with ancestral M1_global_ strains, invasive M1_UK_ strains exhibit reduced genomic diversity and fewer mutations in two-component regulator genes *covRS*. The emergence of M1_UK_ is dated to 2008. Following a bottleneck coinciding with the COVID-19 pandemic, three emergent M1_UK_ clades underwent rapid nationwide expansion, despite lack of detection in previous years. All M1_UK_ isolates thus-far sequenced globally have a phylogenetic origin in the UK, with dispersal of the new clades in Europe. While waning immunity may promote streptococcal epidemics, the genetic features of M1_UK_ point to a fitness advantage in pathogenicity, and a striking ability to persist through population bottlenecks.

## Abstract (web summary)

Exponential growth of toxigenic *Streptococcus pyogenes* M1_UK_ lineage accounted for most of the 2022/2023 invasive infection upsurge in the UK. Authors provide evidence that M1_UK_ first emerged in 2008, has genetic evidence of enhanced fitness, and has disseminated to 3 continents.

## Introduction

Group A Streptococcus (GAS, *Streptococcus pyogenes*) is a human-restricted pathogen causing diseases ranging from sore throat and scarlet fever to more serious invasive infections, including soft tissue infections, pneumonia, and toxic shock, as well as auto-immune sequelae^1^. Although advanced age and specific presentations such as necrotising fasciitis increase the risk of death from invasive infection, the genetic background of *S. pyogenes* strains also contributes to the risk of mortality^2,3^ underlining the role of strain genotype and virulence in disease outcome. Among more than 250 recognised *emm* types, the *emm*1 genotype is most frequently associated with invasive infections in high-income countries^4^. *emm1* strains are considered highly virulent^5,6^ and often acquire inactivating mutations in the *covRS* two-component regulator, which de-represses key virulence factors during invasive infection^7^. In the 1980s, *emm1* emerged as a leading cause of invasive infection following several genomic changes that altered phage content and streptolysin O (SLO) expression, leading to a new clone that spread globally^8^.

In England, prompt notification and antibiotics are advocated for scarlet fever and invasive GAS (iGAS) infections^9^, however guidelines that recommend a non-treatment or delayed treatment approach to sore throat were introduced in 2008, to limit unnecessary use of antibiotics^10^. Unexpectedly large seasonal upsurges in scarlet fever were documented annually in England between 2014-2018^11,12^ coinciding with the expansion and recognition of a new lineage of *emm*1 termed M1_UK_ among *S. pyogenes* isolates^5^. M1_UK_ differed from other globally circulating *emm1* strains^8^ (hereafter referred to as M1_global_) by 27 signature SNPs and was characterised by increased expression of the scarlet fever toxin, streptococcal pyrogenic exotoxin A (*speA*)^5,6,13^. Two intermediate lineages, M1_13SNPs_ and M1_23SNPs_, that share subsets of the 27 SNPs, were also identified^5,6^. M1_23SNPs_ expresses SpeA at the same level typical of M1_UK,_ whereas M1_13SNPs_ does not^6^. By 2016, the M1_UK_ lineage represented 84% of all *emm1* invasive strains in England^5^, increasing to 91.5% by 2020^14^.

The onset of the COVID-19 pandemic, and implementation of non-pharmaceutical interventions (NPI) to limit SARS-CoV2 transmission triggered a reduction in scarlet fever and iGAS notifications in 2020^12^. However, in late 2022, a highly pronounced out-of-season upsurge in both scarlet fever and iGAS cases was reported in England, with unexpected increase in paediatric pleural empyema and several fatalities^15^. Similar increases in severe paediatric iGAS infections were reported worldwide^16^.

In this article, we show that the *S. pyogenes* upsurge in England and Wales was predominantly associated with M1_UK_, a lineage we estimate to have emerged around 2008, and, in particular, three emergent clades that are now widely dispersed. The expansion of M1_UK_ occurred following a bottleneck in growth, likely related to reduced transmission during the COVID-19 pandemic.

## Results

### Trends in *S. pyogenes-*positive samples, England 2016–2023

**Fig. 1:**
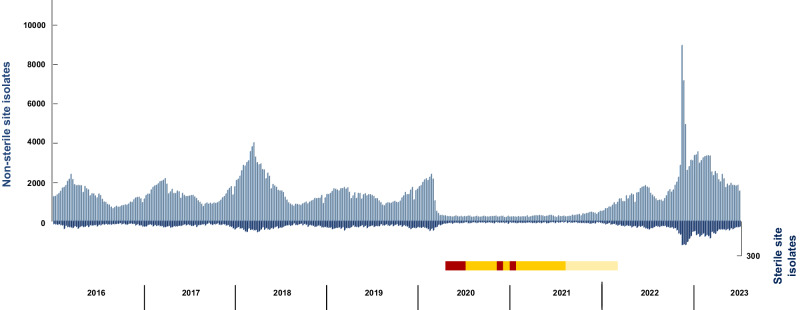
Trend in *S. pyogenes*-positive samples, England 2016–2023. Data show absolute numbers of weekly *S. pyogenes*-positive samples from non-sterile sites (light blue bars, left hand, positive axis) and sterile sites (dark blue bars, right hand, negative axis) recorded by the Second Generation Surveillance System (SGSS) in England, by week and by year. Timing of non-pharmaceutical interventions (NPI) related to COVID-19 in England is indicated by the horizontal bar: red, lockdown periods; orange, legally enforced NPI including no mixing; yellow, non-severe NPI. Schools were closed during lockdown periods and between the two later lockdown periods except for children of key workers and vulnerable children. Source data are provided as a Source Data file Fig. 1.

*S. pyogenes* identified from non-sterile and sterile site samples are recorded through a national laboratory reporting system (Second Generation Surveillance System, SGSS). The typical pattern of seasonal spring-time peaks (Q1-Q2) in *S. pyogenes* infections was interrupted abruptly in April 2020, coinciding with NPI introduced at the onset of the COVID-19 pandemic (Fig. 1). A profound reduction in *S. pyogenes-*positive samples, from both sterile and non-sterile sites, lasted almost two years, ending in Q1 2022. Following cessation of widespread NPI in February 2022, a delayed seasonal increase in microbiologically-confirmed *S. pyogenes* infections returned in April 2022, subsiding only in Q3 2022, in keeping with the UK summer vacation period. Unexpectedly, a second, exponential increase in *S. pyogenes* samples occurred in Q4 of 2022 (Fig. 1). This marked increase in microbiologically-confirmed infections peaked in week 49, when 8906 non-sterile site and 241 sterile site *S. pyogenes*-positive samples were recorded (Fig. 1), coinciding with increased disease notifications^15,17^.

**Fig. 2:**
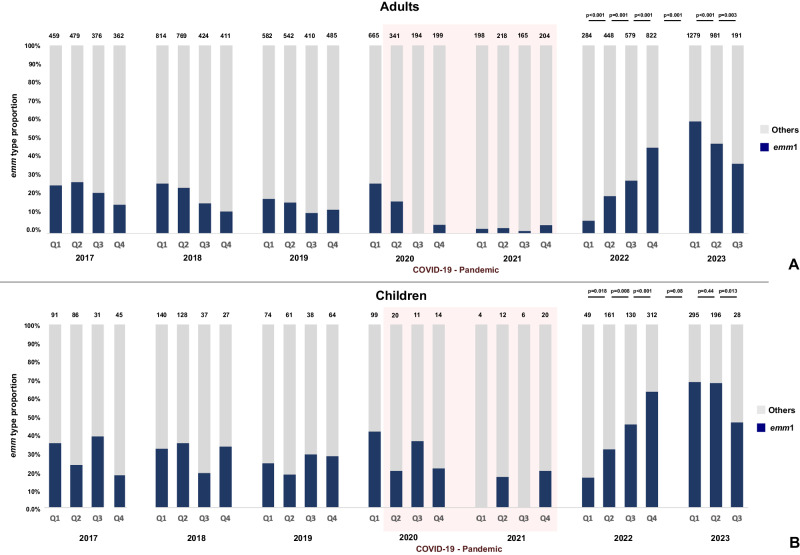
Contribution of *emm*1 *S*. *pyogenes* to invasive group A streptococcal (iGAS) infections 2017–2023. *emm*1 isolates are shown as proportions of the total number of isolates from iGAS cases submitted to and genotyped at the national reference laboratory for each quarter of each year. **A** adults ( ≥ 15 years); **B** children <15 years. The total number of isolates from iGAS cases received by the reference laboratory and genotyped in each quarter are shown on top of each bar; *emm1* proportions are shown in navy blue. Pink shaded region highlights the period of COVID-19 non pharmaceutical interventions. Q1, January-March; Q2, April-June; Q3, July- September; Q4, October-December. Statistical analysis applied to 2022-2023: one-tailed proportion test of *emm1* from Q1 2022 to Q3 2023 (p-values indicated in the figure). Source data are provided as a Source Data file Fig. 2.

*S. pyogenes* isolates cultured from iGAS cases are submitted to the national reference laboratory for *emm* typing. Between Q1 of January 2017 and Q1 of 2020, *emm*1 was the leading cause of iGAS, responsible for 16-28% of all iGAS cases; *emm*1 dominance was greater in children than adults (Fig. 2). During the period of COVID-19-related NPI, annual iGAS isolates reduced ~6.5-fold in children (274 isolates/year 2017-2019; 44 in 2021) and ~2.5-fold in adults (1944 isolates/year 2017-2019; 785 in 2021) (Fig. 2). The proportion of iGAS isolates that were *emm*1 also reduced significantly (p < 0.001), to less than 8% of all iGAS cases. From Q1 of 2022, *emm*1 then showed a sustained quarterly increase in frequency, peaking in Q1 of 2023. For over nine months, *emm*1 accounted for > 50% of all iGAS cases, coinciding with the period of upsurge (Fig. 2). Indeed, *emm*1 was the only genotype to expand significantly during this time, increasing from 20% to 55%. In children ( < 15 years), this increase was more apparent; *emm*1 accounted for 60% and 70% of iGAS in the same period (Fig. 2).

### Population genomics of *emm*1 *S. pyogenes* strains comprising the upsurge

**Fig. 3:**
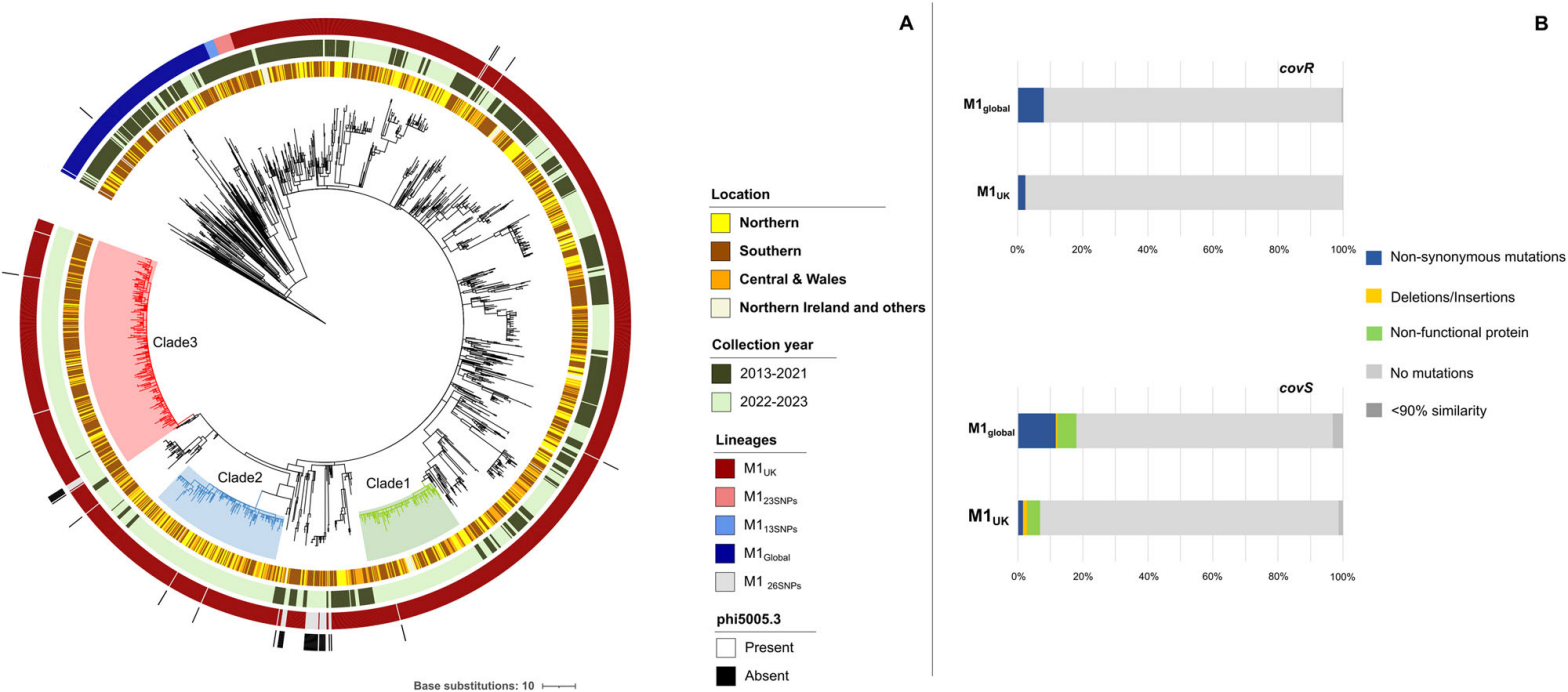
Genetic analysis of 1815 *emm1 S. pyogenes* isolates from invasive group A streptococcal (iGAS) infections 2013-2023. **A** Phylogenetic tree comprising sequenced *emm1* isolates associated with invasive infections (iGAS) from 2013-2023 sequenced at reference laboratory: Maximum likelihood phylogenetic tree constructed from 278 core SNPs (excluding recombination regions) extracted after mapping 1815 *emm1* isolates to the MGAS5005 reference genome. The tree was drawn in a circular layout and rooted on outgroup genome NCTC8198. Bars in concentric circles represent (from inside to outside) regional location of isolate; collection period (pre-upsurge 2013-2021 or upsurge 2022-2023); *emm*1 lineage, and presence/absence of the phi5005.3 phage. Regional data have been grouped for purpose of data visualisation as follows: Northern (North-East England, North-West England, Yorks & Humber); Central and Wales (East Midlands, West Midlands, Wales); Southern (South-East England, South-West England, London); and Northern Ireland and others (comprises regions with less than 5 isolates including Scotland, Eire, Jersey, Malta). **B** Frequency of *covR* and *covS* non-synonymous and other mutations within M1_UK_ and M1_global_ isolates from invasive infections. Percentage of strains with non-synonymous mutations, deletions/insertions, or an inactive protein in 1552 M1_UK_ and 189 M1_global_ isolates is shown. Mutation types are indicated by coloured bars. Percentage of strains where sequence quality precluded analysis (sequence identity <90%) are in dark grey. Differences in *covR* and *covS* mutation frequency between M1_global_ (covR 15/189; *covS* 34/189) and M1_UK_ (*covR* 38/1552; *covS* 106/1552) are significant (one-tailed proportion test: *covR* p < 0.001; *covS* p < 0.001)_._ Ten M1_global_ isolates formed a previously unrecognised clade with covRS mutations. If all strains from this cluster are removed, the *covS* mutation frequency within M1_global_ (24/179) remains significantly greater than M1_UK_ (106/1552) strains (one-tailed proportion test covS p < 0.001). Source data are provided as a Source Data file Fig. 3.

To investigate any genetic basis for the increase in *emm*1 iGAS cases, genomes of all 1092 iGAS *emm1* isolates submitted to the reference laboratory from January 2022 to March 2023 were whole genome sequenced. Phylogenetic analysis revealed clustering of *emm*1 genomes into expected lineages. The vast majority (1001/1092, 91.8%) of isolates were M1_UK_, 4.1% (44/1092) were derivatives of M1_UK_ having lost the phi5005.3 phage (and therefore lacking the phage portal protein SNP that is typical but not essential to M1_UK_) and 4.2% (46/1092) were M1_global._ Taken together, 95.7% of all *emm*1 strains from the 2022/2023 upsurge period were M1_UK_ or a 26SNP derivative thereof, representing overall expansion of the lineage since 2020^14^ (Fig. 3A). Isolates from 2022/2023 were further compared to 723 *emm*1 iGAS strains sequenced in the same reference laboratory between 2013-2021 to determine evidence for recent genomic change. Phylogenetic analysis of these 1815 *emm*1 *S. pyogenes* genomes associated with iGAS showed M1_UK_ isolates from 2022/2023 to be broadly distributed across the pre-existing M1_UK_ population, with three emergent dominant clades and several small clades formed almost exclusively of isolates from 2022/2023 (Fig. 3A). Three clades accounted for over half (54.8%) of all M1_UK_ from 2022/2023. Clade 1 comprised 123 invasive strains exclusively from 2022/2023 and was characterised by two SNPs (Supplementary Table 1). Clade 2 comprised 166 invasive strains exclusively from 2022/2023 and was characterised by 6 SNPs, including three non-synonymous mutations (in *sic*1.01, *pyrC* and M5005_Spy1146). Clade 3 comprised 284 strains from 2022/2023, plus a single strain collected in February 2020, and was defined by 3 non-synonymous mutations (in *xerD*, *huTu* and *secA*). Clade 3 was enriched by invasive strains collected in southern England (70%), consistent with regional transmission. In contrast, Clades 1 and 2 had similar proportions of strains from northern (26% and 35%), southern (43% and 35%), and central regions including Wales (23% and 28%) consistent with a wider national outbreak (Fig. 3A). The average genetic distance between any two strains from Clade 1 was just 2 SNPs, while for Clades 2 and 3, the average was just 3 SNPs (Supplementary Table 2). The low diversity was consistent with rapid emergence and dispersion through the year and across the country from a recent common ancestor.

Among the 1815 *emm*1 genomes associated with iGAS from 2013-2023, the clinical sources of isolates were known for most strains: 67.7% (1229/1815) were blood isolates; 6.9% (125/1815) were lower respiratory tract isolates, of which 71.2% (89/125) were pleural sample isolates, indicative of empyema (Supplementary Table 3). Overall, a higher proportion of M1_UK_ (5.0%) isolates were associated with pleural samples compared to M1_global_ (2.6%), in particular Clade 3 (8.4%) (Supplementary Table 4). Considering only diseases occurring in 2022/2023, inter-lineage differences were not significant, however M1_global_ isolate numbers were very low (Supplementary Table 4). Pleural sample isolates were notably more frequent at the time of the upsurge. Despite the notable impact of the upsurge on children, no single clade was uniquely associated with a specific age group, and closely related strains ( < 3 SNPs apart) caused invasive infections in both adults and children (Supplementary Fig. 1).

The average pairwise distance within M1_UK_ increased from 16 SNPs in 2013-2021 to 22 SNPs in 2022/2023, while the average pairwise distance within the M1_global_ lineage increased from 39 SNPs in 2013-2021 to 55 SNPs in 2022/2023 (Supplementary Table 2). Despite the recent increase in the genetic diversity of both lineages (M1_global_ and M1_UK_), M1_UK_ showed greater genomic stability (point mutations) than M1_global_. Most mutations (excluding the 27 M1_UK_ signature SNPs) were unique to individual strains outside the main clades (Supplementary Fig. 2) consistent with a rapid population size expansion. The four indels previously reported^13^ were present in 99% of M1_UK_ isolates but were not lineage specific (Supplementary Data 1).

Recombination and pangenome analyses showed little evidence of gain or loss of transferable elements between M1_UK_ and M1_global_, and no genomic feature(s) associated only with M1_UK_ from 2022/2023 or M1_global_ from 2022/2023, or the three M1_UK_ clades previously described. Most strains had three prophages typical of *emm*1: Φ5005.1, which encodes *speA*; Φ5005.2, which encodes *spd3* or *spd4*; and Φ5005.3, which encodes another DNase, *sdaD2/sda1*, reported to contribute virulence to modern M1_global_ strains^8^. Although M1_UK_ strains are characterised by increased SpeA expression, 9/1552 (0.6%) invasive M1_UK_ strains had a partial deletion of phage Φ5005.1 including *speA* (Supplementary Table 5). Furthermore, 43/1552 (2.8%) invasive M1_UK_ strains had lost Φ5005.3 and consequently cannot express *sdaD2/sda1*. Prophage Φ370.1 containing *speC* and *spd1* was present in ~10% (174/1815) of *emm*1 strains, 9% (139/1552) in M1_UK_, and 16% (31/189) in M1_global_. Only 4/1815 *emm*1 strains (one M1_global_ and three M1_UK_) from 2014-2020 had the ΦSP1380.vir phage (with *speC, ssa, spd1*) reported in Australia^13^ and Hong Kong^18^.

*Emm*1 invasiveness has been associated with regulatory gene mutations in vivo^7^. Among iGAS clinical isolates from invasive infection, mutations in the two-component regulatory genes *covR* and *covS* were significantly more frequent in M1_global_ (7.9% and 18% respectively) than M1_UK_ (2.4% and 6.8%) (one-tailed proportion test: *covR* *p-*value 0.001; *covS* *p*-value < 0.001) (Fig. 3B), pointing to greater selection pressure on M1_global_ strains during invasive infection. Though observed in both sterile and non-sterile site isolates from invasive infections, this difference in the frequency of *covS* mutations could not be replicated by in vivo passage of non-invasive M1_global_ and M1_UK_ isolates in mice, although only five strains from each lineage were tested using intramuscular inoculation (Supplementary Fig. 3). Mutations in *rgg*1 and *rgg*4 were frequent, but not different between lineages (Supplementary Fig. 4). The frequency of resistance to common antimicrobials among *emm*1 isolates was low ( < 0.5%); furthermore, *pbp2x* missense mutations (T553K and P601L)^19,20^ were absent in our dataset (Supplementary Data 2).

### Relationship between non-invasive and invasive *emm*1 isolates

**Fig. 4:**
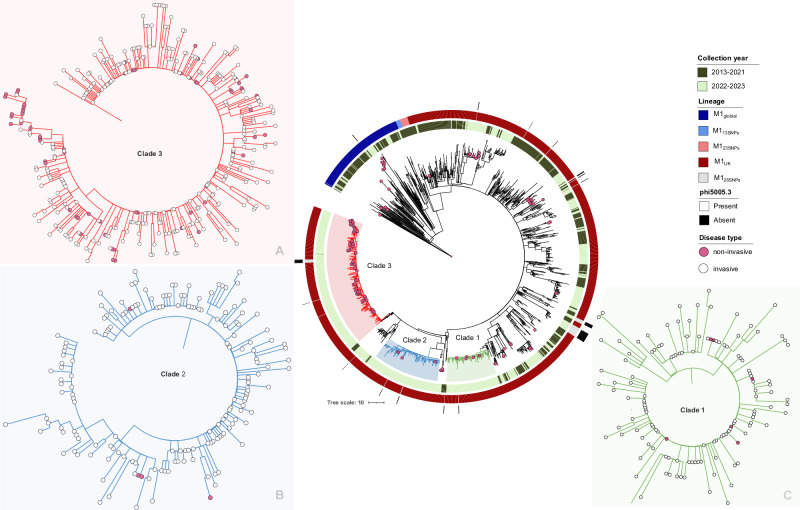
*emm1* phylogenetic tree showing non-invasive sore throat isolates collected in London in 2022 with isolates from invasive infection from UK 2013–2023. Maximum likelihood phylogenetic tree constructed with the core alignment of 274 SNPs extracted after mapping 1815 *emm1* invasive isolates and 133 non-invasive isolates against MGAS5005. The relationship between invasive and non-invasive infection isolates within Clades1-3 is shown in inset (**A**) Clade 3; (**B**) Clade 2; and (**C**) Clade 1. Source data are provided as a Source Data file Fig. 4.

To extend our analysis to include the reservoir of non-invasive pharyngitis *S*. *pyogenes* isolates, we sequenced 133 *emm*1 strains collected sequentially from pharyngitis cases in west London in 2022-2023. 14.3% (19/133) of *emm*1 throat isolates were M1_global_ while 85.7% were either M1_UK_ (111/133) or M1_UK_ without the phi5005.3 phage (3/133). Interestingly, the proportion of non-invasive and invasive M1_global_ isolates was higher in London than observed nationally during the same period. Phylogenetic analysis of invasive and non-invasive isolates showed that non-invasive M1_UK_ isolates from west London clustered mostly within Clade 3 (62/111, 55.9%), with other isolates scattered throughout the wider M1_UK_ population, including Clade 1 (8/111, 7.2%) and Clade 2 (4/111, 3.6%) (Fig. 4). The average number of mutations between two isolates from the same clade (Clade 1, 2 or 3) was 2-3 SNPs. 48% (64/133) of non-invasive isolates were found to be identical to at least one invasive isolate (0 SNPs apart, Fig. 4). Point mutations in bacterial regulatory genes in non-invasive *emm*1 sore throat isolates were rare ( < 5%), in comparison to invasive isolates. 5/133 (4%) of non-invasive isolates collected in London in 2022 had the ΦSP1380.vir phage.

### Time and place of emergence of M1_UK_ and intermediate lineages

**Fig. 5:**
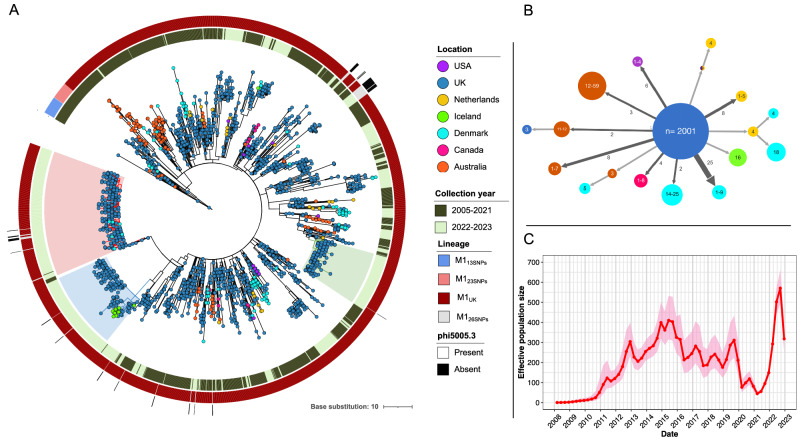
Global distribution and potential introduction events of M1_UK_ and intermediate populations. **A** Phylogenetic tree of 2364 M1_UK_ and intermediate strains collected globally March 2005 to July 2023. The tree was built based on 3406 SNPs from a core genome alignment relative to M1_UK_ (H1490/NCTC14935) reference genome and rooted on a closely related M1_global_ genome gas81595 (also included in this tree). Leaves are coloured based on the country where samples were collected. Shading indicates the 3 emergent clades (Clade 1, green; Clade 2, blue; Clade 3, red). Coloured bars in concentric circles represent (from inside to outside): collection years (pre-upsurge 2013-2021 and upsurge 2022-2023); *emm*1 lineage; and presence/absence of the phi5005.3 phage. **B** Simplified transmission tree by PastML showing the ancestral epidemic location of M1_UK_ and intermediate lineages. Each node represents a cluster of leaves sharing the same probable ancestral location and is labelled by the range of leaves numbers. Each arrow indicates inferred international transmission events; arrow width and labels indicate the number of identical origin-destination transmission events. For example, the arrow labelled “6” pointing at the node “1–4” (USA) indicates six clusters of 1 to 4 leaves were present in the USA that were likely imported from the UK. **C** Estimated effective population size (*N*_*e*_) of M1_UK_ in the UK through time. The red line and pink shading at each time point indicate the mean and 95% confidence interval of *N*_*e*_, respectively. Source data are provided as a Source Data file Fig. 5.

To elucidate the origin and time of emergence of the M1_UK_ lineage, a dated phylogenetic tree was constructed using a newly sequenced M1_UK_ reference strain H1490 (NCTC14935). The tree comprised 2364 M1_UK_ and intermediate (M1_13SNPs,_ M1_23SNPs_ and M1_26SNPs_) genomes collected from Europe (Denmark^21^, Iceland^21^, Netherlands^22^), United Kingdom^5,23^, plus the isolates from the current study, North America (Canada^24^ and USA^25^), and Australia^13^ between March 2005 and July 2023. This showed M1_13SNPs_ and M1_23SNPs_ to share a common ancestor with the M1_UK_ lineage, while M1_26SNPs_ are derivatives of M1_UK_ that have lost the Φ5005.3 phage (Fig. 5A). According to the inferred ancestral dates in the tree, the M1_13SNPs_ lineage diverged in 2002 (95% confidence interval (CI): 2000–2004), followed by M1_23SNPs_ in 2006 (95% CI: 2004–2007), and M1_UK_ in 2008 (95% CI: 2006–2009), prior to rapid expansion. The genome-wide mutation rate was estimated to be 1.49 nucleotide substitutions per year.

Ancestral state reconstruction of geographical locations was limited to those regions that undertake and report sequencing of *S. pyogenes*; this revealed that M1_UK_, M1_13SNPs_ and M1_23SNPs_ originated in the UK and then dispersed, with multiple independent introductions into Australia, North America, Netherlands, Iceland, and Denmark (Fig. 5A-B). Denmark and UK strains collected in 2022-2023 were dispersed within the M1_UK_ circulating population, including Clade 3, while almost all 2022-2023 Iceland isolates grouped together in Clade 2.

Bayesian inference of the M1_UK_ effective population size through time in the UK demonstrated rapid population growth of M1_UK_ from 2008 until 2015, followed by a progressive decline until 2019, and then a sharp decline in early 2020 (Fig. 5C). Strikingly, the population dynamics suggested a transmission bottleneck in M1_UK_ during the implementation of severe NPI designed to limit the spread of COVID-19 (April 2020 –March 2021). The mean effective population size over this period dropped to one-fifth of the pre-pandemic maximum and then rose steeply after the lifting of the lockdown and other NPI measures. Importantly the inferred patterns of population growth and decline were not driven by any variation in the number of sequenced M1_UK_ isolates in the UK through time (Supplementary Fig. 5).

## Discussion

The marked increase in bacteriologically confirmed *S. pyogenes* infections in England in late 2022-2023 coincided with the reported national upsurge in notifications of both scarlet fever and iGAS^15,16^. The upsurge in invasive infections was clearly associated with a significant increase in *emm*1 *S. pyogenes* only, the vast majority (95.7%) of which belonged to the emergent M1_UK_ lineage or its derivatives. No substantial genomic changes in M1_UK_ were observed during the upsurge, but three new clades emerged and expanded within M1_UK_, accounting for 53% of *emm*1 iGAS in 2022-2023.

Several countries have now reported similar iGAS upsurges in the period 2022-2023, chronologically associated with the end of mitigation strategies implemented during the COVID-19 pandemic^21,26–29^, including association with *emm*1^26^ or M1_UK_^28,29^. Although *emm*12 infections were prominent in early 2022^15^ as reported elsewhere^29,30^, the very marked increase in iGAS observed in the second half of 2022 in England was accounted for by *emm*1. The dominance of *emm*1 among invasive isolates ( > 50% overall, and almost 70% in children) is unprecedented in UK records. In contrast, during the period of the COVID-19 pandemic-related NPI in 2020-2022, bacteriologically confirmed *S. pyogenes* infections were rare. While reduction in non-invasive infection detection might be explained by a reduction in consultations, this would not explain the reduction in sterile site isolates. Furthermore, during the period of COVID-19 NPI, invasive infections due to *emm*1 were exceedingly rare, with no *emm*1 isolates identified during some quarters of 2020-2021 in either adults or children. We posit this points to differential modes of transmission, whereby ‘throat specialist’ strains^31^ such as *emm*1 require respiratory transmission in order to circulate, while others may spread via skin contact.

The reported increase in iGAS in late 2022 was particularly evident in children, with complicated clinical presentations including meningitis^28^ and, specifically, rapidly progressive pleural empyema in countries where such data are collected^15,29^. Isolates from empyema are often not cultured due to antibiotic pre-treatment. Hence, the pleural sample isolates in the current study represent a subset of all pleural empyema cases. Regardless, pleural isolates were significantly associated with the emergent M1_UK_ clades. The timing of the upsurge in Q3 2022 is very likely to have contributed to the pleural empyema phenotype; respiratory viral infections were identified in 25% of paediatric cases of empyema^15^, playing a potential role in progression to lower respiratory tract infection. Due to the design of our study and the widespread adoption of respiratory viral point-of-care tests to diagnose respiratory viral infection in 2022, we are unable to assess the effect of respiratory viral infection as a contributor to empyema over time in the current study.

M1_UK_ is increasingly dominant in the UK. Our findings are mirrored to different degrees in other countries, where the proportion of *emm*1 isolates that are M1_UK_ ranges from 41.5%-78%^21,28,29^. The fitness of M1_UK_ has been attributed to its ability to express SpeA, a superantigen that can promote pharyngeal infection^5^. Increased SpeA is associated with a SNP in the leader sequence of *ssrA*^13^, which is present in not only M1_UK_ but also the near-extinct intermediate M1_23SNPs_ lineage. The contraction of the M1_23SNPs_ lineage suggests that additional fitness advantages prevail in M1_UK_^6^. Genome stability appeared greater in M1_UK_ than M1_global_, suggesting the accumulated 27 SNPs in M1_UK_ may be sufficient to confer a fitness advantage during human infection, including increased transmissibility. Indeed, in one study, the mean secondary attack rate was 40% among asymptomatic contacts of M1_UK_ infection in two classes of schoolchildren, compared with 22.8% in classroom outbreaks involving different *emm* types^32^, supporting a potential transmission advantage. In the current study, M1_UK_ invasive isolates were significantly less likely to exhibit mutations in *covRS* than M1_global_ strains, suggesting a fitness advantage in invasive infection as well. Although we were unable to reproduce this difference experimentally, the intramuscular route of infection in mice does not reflect the bottleneck of natural mucosal infection in humans and was necessarily limited to just five strains per group.

A comparison of non-invasive *emm*1 isolates from London and invasive *emm*1 isolates nationally revealed both groups to be interspersed and clustered tightly in the phylogenetic tree, indicating a common genetic pool. The analysis showed that individual invasive isolates can be derived repeatedly from the population of pharyngitis strains. The identical nature of strains underlines the route of direct transmission from cases of pharyngitis and scarlet fever to dangerous invasive infections, often unnoticed. We found that diversifying selection in the invasive population, especially in M1_global_, drives the accumulation of mutations in *covRS*, as reported^33^.

Our study evaluated the origin, dispersion, and population dynamics of M1_UK_ by assembling the most comprehensive global collection of M1_UK_ strains to date. The analysis showed M1_UK_ to be globally distributed, with nearly identical strains found all over the world and multiple introductions from the UK population. The 2022 upsurge in the UK was characterised by the rapid expansion of three clades within M1_UK_, of which two showed swift dispersal to at least two other European countries. In Iceland, a single introduction event appeared responsible for reported M1_UK_ cases, whereas in Denmark, multiple introductions seemed likely. We found no evidence of importation of a new lineage recently reported in Denmark (M1_DK_)^21^.

The origin of M1_UK_ was estimated to date from 2008, the year in which national guidelines to reduce swab testing and unnecessary antibiotic treatment of sore throat were introduced in England^10^. An exponential increase in the M1_UK_ population commenced around 2010. Given the propensity for M1_UK_ to spread readily in classrooms^32^, it is conceivable that new lineages can emerge and rapidly expand if active *S. pyogenes* throat infections are not detected and treated with antibiotics and transmission is not controlled. Antecedent intermediate lineages emerged in 2002 (M1_13SNPs_) and 2006 (M1_23SNPs_), during which time secular changes in sore throat management were ongoing in the UK^34,35^.

Our dataset is limited to the UK and other high-income temperate countries, hence no inferences about the importation of M1_UK_ into low-income countries were possible. This underlines the importance of global surveillance to monitor the evolution and epidemiology of emerging variants with increased capacity for pathogenicity. Although M1_UK_ geographic origin was identified as the UK, this was the only country with genomes available from the time of emergence, as such, we cannot exclude an alternate origin.

The phylodynamic analysis of M1_UK_ in the UK showed a decline in population size between 2015-2019 after the initial rapid rise, consistent with the cyclical changes in *S. pyogenes* populations known to occur^36^, however population size plummeted in early 2020 when NPI to combat spread of COVID-19 were introduced. The marked M1_UK_ population bottleneck was followed by rapid expansion in 2022 and 2023, raising the question of whether strain-specific survival advantages exist during periods of such low *S. pyogenes* population activity. Global reductions in other bacterial respiratory pathogens were seen during the period of COVID-19 NPI^37^. However, the scale of resurgence in invasive *S. pyogenes* following the relaxation of NPI thus far appears unique, perhaps related to the lack of a vaccine for *S. pyogenes* compared with other pathogens studied^37^. The observed magnitude and severity of the upsurge could be explained by the coincidence of enhanced M1_UK_ pathogenicity and diminished human population immunity to *S. pyogenes*, as a predictable but perhaps unintended consequence of interventions to limit the spread of COVID-19^38^. The role of exposure-driven human immunity in shaping cyclical and post-COVID-19 changes in *S*. *pyogenes* epidemiology is the subject of ongoing research. Scarlet fever affects children in their first year of school^39^, an experience that was delayed for many during two years of COVID-19-related NPI. We hypothesise this resulted in a ~ 3-fold increase in susceptible children starting school in Q3 2022, with a similar reduction in immunity in siblings and adults. We posit that the transmissibility and invasiveness of M1_UK_ facilitated the exponential and unprecedented increase in invasive *S*. *pyogenes* infections.

## Methods

### Surveillance of *S. pyogenes* detection in clinical samples in England

UK Health Security Agency surveillance of infections for health protection purposes is approved under Regulation 3 of The Health Service (Control of Patient Information) Regulations 2020 and under Section 251 of the NHS Act 2006. All reports of *S. pyogenes-*positive clinical samples, including post-mortem, from ISO-8601 week 1 2016 to week 30 2023 reported by English laboratories were extracted from the UK Health Security Agency (UKHSA) Second Generation Surveillance System (SGSS) on 7 December 2023. SGSS captures approximately 98% of electronically supplied hospital microbiology laboratory data in England; however, is the primary route for statutory reporting^40^ of laboratory-confirmed invasive *S. pyogenes* infections. Invasive *S. pyogenes* samples are defined as culture-positive samples (or positive by molecular detection) obtained from a normally sterile site. *S. pyogenes-*positive samples were deduplicated where patients had more than one positive *S. pyogenes* similar specimen type taken on the same date.

### Invasive Streptococcus pyogenes isolates

*S. pyogenes* isolates from invasive disease (iGAS) cases in England, Wales, and Northern Ireland are routinely submitted to the national reference laboratory (SSRS, Staph and Strep Reference Section, UKHSA, London, UK) for *emm* genotyping using standard methods (https://www.cdc.gov/streplab/groupa-strep/emm-typing-protocol.html). Processes and reporting requirements for isolate submission, including clinical sample source, were unchanged during the study period. The percentage of invasive isolates that were determined to be *emm*1 was determined compared with the overall total number of isolates genotyped. As part of the investigation into the upsurge of *S. pyogenes*, all *S. pyogenes* isolates from 2022/23 were whole genome sequenced (WGS). For this study, we included all *emm*1 isolates from invasive infections that had been genome sequenced at the reference laboratory from 2014–2023, including a small number from other regions. This included *emm1* isolates from 2014-2015, previously reported (*n* = 516)^41^; *emm1* isolates from 2016–2021 (*n* = 207) intermittently sequenced as part of service delivery; and all *emm1* strains (*n* = 1092) submitted to the reference laboratory from January 2022-March 2023 that were sequenced as part of this outbreak investigation. Metadata and accessions for all isolate genome sequences are listed in Supplementary Data 3. Isolate WGS was linked to reported clinical sample type. Differences in the proportion of *emm*1 between time points were evaluated using a one-tailed proportion test (https://www.socscistatistics.com/tests/ztest/).

### Non-invasive *S. pyogenes* isolates

The collection and analysis at Imperial College London of fully anonymised bacterial isolates from a diagnostic laboratory previously linked to routine data was approved by a national research ethics committee (West London Research Ethics Committee 06/Q0406/20). *S. pyogenes* throat isolates were identified by MALDI-Biotyper (Bruker) from swabs submitted to the Diagnostic Laboratory at Imperial College Healthcare NHS Trust (London, UK) during 2022 (1 January - 31 December). This laboratory serves northwest London, a population of ~2 million people, representing ~3.5% of the population of England. *S. pyogenes* isolates were cultured on Columbia Blood Agar (CBA, Oxoid, Basingstoke, UK) or in Todd Hewitt broth (Oxoid) at 37 °C with 5% CO_2_. Demographic data were linked to all isolates and anonymised in accordance with the approved protocol (06/Q0406/20). All *emm*1 pharyngitis isolates (from throat swabs) were genome sequenced at the National Reference Laboratory (Supplementary data 3).

### Genomic data contextualisation

Three different genomic datasets were included in this study. The first contains 1815 (1092 newly sequenced from 2022-2023; and 723 from 2013-2021) *emm*1 strains associated with invasive infections collected at the national level and sequenced at the UKHSA national reference laboratory from 2013 to 2023 (Supplementary Data 3); 12 isolates were from outbreak investigations. The second dataset contained the 1815 invasive strains described above plus 133 newly sequenced non-invasive *emm*1 isolate whole genome sequences (WGS) collected in London during 2022 as part of this study (1 January to 31 December), yielding a total of 1948 *S. pyogenes* isolate WGS (Supplementary Data 3). The third dataset was created to provide phylogenetic context for the M1_UK_ global population and intermediate strains only. This dataset included an additional 385 previously-published M1_UK_ or intermediate WGS from the UK sequenced at the Wellcome Trust Sanger Institute dating from 2005-2018^5,23^; 163 M1_UK_ or intermediate WGS collected in Australia 2010-2022^13^; 16 M1_UK_ or intermediate WGS collected in Canada 2016-2019^24^; 120 M1_UK_ or intermediate WGS collected in Denmark 2018-2023^21^; 18 M1_UK_ or intermediate WGS from Iceland, 2023^21^; 27 M1_UK_ or intermediate WGS collected in the Netherlands 2019^22^; and 10 M1_UK_ or intermediate WGS from USA collected in 2015-2018^25^. Data collection finished in July 2023, and therefore genomes reported after that time point were not included. The final global dataset contained 2365 M1_UK_ and intermediate strains (Supplementary Data 3).

### Generation of new M1_UK_ reference genome: Reference strain NCTC14935

Genomic DNA from *S*. *pyogenes* M1_UK_ isolate H1490 and M1_global_ isolate H1499 (both sore throat isolates) was sheared using a Megaruptor to prepare 20-22 kb PacBio SMRT libraries, following the manufacturer´s recommendations. The libraries were sequenced using one Single Molecule Real-Time (SMRT) cell in a PacBio RSII platform (Pacific Biosciences of California, Inc., Menlo Park, CA, USA) at the University of Edinburgh. The data was demultiplexed using Lima v2.2.0 (https://lima.how/). The demultiplexed CLR data was converted to CCS using ccs tool v6.3.0, and further HiFi reads (CCS > Q20) were extracted using extract hi fi tool from the same package. The genome assemblies were generated from the HiFi reads using Redbean v 2.25^42^ and Trycycler v0.5.3^43^. The assembly quality was assessed using QUAST v5.0.2^44^ and BUSCO v5.3.0^45^. The annotation was performed using prokka v1.14.6^46^. PacBio sequencing reads and data are deposited in the European Nucleotide Archive under BioProject accession PRJEB68198 (M1_UK,_ H1490 - ERR12378139 and M1_global,_ H1499 - ERR12378140). The two isolates have been deposited in the National Collection of Type Cultures (NCTC) with the accessions NCTC14935 (M1_UK_, H1490) and NCTC14936 (M1_global_, H1499).

### Illumina genome sequencing, assembly, and annotation

For this study, whole genome sequencing of all clinical isolates (invasive and non-invasive) was performed by the UKHSA reference laboratory using the Illumina NextSeq 1000 platform with 100 base paired-end chemistry. Reads were trimmed to remove adaptor sequences and low-quality bases with Trimmomatic v0.39^47^. Contamination was assessed based on Kraken2^48^ classification of reads mapped against a standard database for bacteria. Genomes with less than 90% of the reads mapped against *S*. *pyogenes* were excluded. Draught genomes were generated using SPAdes v3.15.4^49^. The assembly quality was assessed using QUAST v5.0.2^44^, and poor assemblies were filtered out if the genome size was higher than 2.1 Mbp and/or had more than 400 contigs. Genome annotation was performed with prokka v1.14.6^46^.

### Identification of single nucleotide variations and phylogenetic analysis

Core genome alignment was obtained by mapping trimmed reads of *S*. *pyogenes* genomes to MGAS5005 (GenBank accession: CP000017.2) reference genome using snippy v4.6.0 (https://github.com/tseemann/snippy), with a minimum coverage of 10, a minimum fraction of 0.9 and minimum vcf variant call quality of 100. The SNP distance matrix was obtained using snp-dist (https://github.com/tseemann/snp-dists). SNPs identified were classified as non-coding, missense or synonymous according to their location in the genome and their effect on protein sequence using Snippy. Gubbins v3.3.0^50^ was used to identify and remove recombinant regions. A maximum-likelihood (ML) phylogenetic tree was constructed from the multi-sequence alignment using RAxML-NG v1.0.1^51^ implemented in Gubbins v3.3.0 (substitution and rate variation model: GTR + Gamma). The ML tree was rooted on NCTC8198 (GenBank accession: GCA_002055535.1, reference genome of old *emm1* lineage). Phylogenetic trees and associated data were visualised using iTOLv6.8.1^52^.

### Characterisation of genomic features of interest

The presence of AMR genes was predicted by combining the results from ABRicate (https://github.com/tseemann/abricate), Ariba^53^ and srst2^54^. The *pbp* gene sequences (*pbp1a*, *pbp1b*, and *pbp2x*) were obtained using a BLASTN (NCBI BLAST+ v2.7.1) search. The nucleotide sequences were converted to amino acids and examined for the presence of non-synonymous mutations. None of the non-synonymous mutations previously associated with penicillin resistance in *S*. *pyogenes* were identified. A similar approach was used to identify non-synonymous mutations in *S*. *pyogenes* regulatory genes (*covR*, *covS*, *fasA*, *fasB*, *fasC*, *rgg1*, *rgg2*, *rgg3*, *rgg4*, *rivR*, *rofA* and *rocA*). The presence of superantigens (*smeZ*, *speA2*, *speC*, *speG*, *speH*, *speI*, *speJ*, *speK*, *speL*, *speM*, *speN*, *speO*, *speP*, *speQ*, *speR*, *ssa*) and DNAses (*sda2*, *sdn1*, *spdn1*, *spd3*, *spd4*, *spdB*, *spnA*) was accessed with a BLASTN (NCBI BLAST+ v2.7.1) analysis with the default parameters. Differences between lineages (M1_global_ and M1_UK_) regarding the number/type of mutations found in regulatory genes and *pbp* genes were evaluated using a one-tailed proportion test (https://www.socscistatistics.com/tests/ztest/). Regulatory gene sequences with <90% similarity to the reference genome were excluded from the identification of regulatory gene mutations.

### Pangenome analysis

A pangenome graph was constructed from annotated genome assemblies of MGAS5005 and 1815 *emm1* isolates collected from across the UK between 2013 and 2023 using Panaroo v1.3.0^55^ under its moderate decontamination mode. Clusters of orthologous genes (COGs) were defined by a minimum nucleotide identity of 98%, and core genes were defined by a minimum frequency of 95%. The resulting gene presence-absence matrix was filtered to remove pseudo and fragmented genes as well as those of unusual lengths. The pangenome graph was simplified with the MGAS5005 genome as a reference using Panaroo’s helper script *reference_based_layout.py* for visualisation in Cytoscape v3.10.1^56^. Presence-absence of COGs was compared between M1_UK_ and M1_global_ and between pre-2022/2023 and 2022/2023 groups using Python v 3.11.6.

### Phylodynamic analysis of M1_UK_

A maximum-likelihood (ML) phylogenetic tree corrected for recombination events was constructed from the multi-sequence alignment of global M1_UK_ and intermediate genomes (against the M1_UK_ reference genome H1490) using RAxML-NG v1.0.1^51^ as implemented in Gubbins v3.3.0^50^ (model: GTR + Gamma). The ML tree was rooted on M1_global_ isolate gas81595 (ERS17508611), which was the most closely related to M1_UK_ and intermediate lineages according to SNP distances. A dated phylogenetic tree was generated from the ML tree using the least-squares dating method implemented in the LSD2 module of IQ-Tree v2.2.2.7 (model: GTR + I + G4)^57,58^. Ancestral geographical locations were inferred from the dated tree and isolate information using the MPPA method and F81 model as implemented in PastML^59^.

To reconstruct the population dynamics of the M1_UK_ lineage in the UK, a UK-specific subtree of M1_UK_ genomes was extracted from the dated tree, and the M1_UK_ effective population size (N_e_) was thereby modelled through time using a skygrowth model^60^ implemented in R package mlesky (with 60-time intervals as determined using the package’s parameter-optimisation algorithm based on the Akaike Information Criterion)^61^. Furthermore, the same model was iteratively fitted on 40 subtrees of randomly sampled UK M1_UK_ genomes (with a maximum of 76, 22, and 14 genomes per year, respectively, based on sample sizes between 2019 and 2021) to evaluate if the variation in sample size over time could impact the inference of N_e_.

### In vivo screening for *covR/S* mutations using five representative strains of M1_UK_ and M1_global_

All animal experiments were undertaken using protocols approved by the Imperial College Animal Welfare Advisory Board (AWERB) and authorised by a UK Home Office Project Licence. Mice were maintained in a standard 12 h light/12 h dark cycle with food and water available ad libitum.

Five M1_global_ and five M1_UK_ strains were used in this study (Supplementary Fig. 3). Strains were selected from isolates from 2022 that were broadly representative of each lineage and that had no existing *covR* or *covS* mutations. Experimental soft tissue infections were performed using female BALB/c mice aged 6 weeks (Charles River, UK). Bacteria were cultured on CBA overnight and resuspended in sterile PBS. Mice were infected with 5 × 10^8^ CFU of one of the 10 strains (3 mice per strain) into the thigh muscle. 24 h after infection, mice were sacrificed, and 150 μl heparinized blood obtained by cardiac puncture from each mouse was plated onto CBA prior to euthanisation. Each spleen was removed, homogenised using FastPrep-24™ 5 G in 1 ml PBS and plated on CBA for enumeration. Agar-based casein digestion assay was used to determine SpeB activity to infer *covS* mutations. 50 colonies cultured on CBA from spleens were patched onto 2% w/v skim milk Todd Hewitt agar (THA) to determine SpeB activity. One spleen sample with only a single colony was excluded from analysis; three samples with 16, 33 and 36 colonies were included. Fifty colonies from the inoculum of each strain were patched onto skim milk THA to rule out *covS* mutations occurring before introduction to the mice. SpeB (caseinolytic) activity was determined by comparing zones of clearance from *S*. *pyogenes* isolates to positive controls on the same plates and repatched to confirm the phenotype. Statistical analysis was performed with GraphPad Prism 10. A comparison of the two groups was carried out using a two-tailed nested t-test.

### Reporting summary

Further information on research design is available in the Nature Portfolio Reporting Summary linked to this article.

### Supplementary information

Supplementary Information

Peer Review File

Description of additional supplementary files

Supplementary Data 1

Supplementary Data 2

Supplementary Data 3

Reporting Summary

### Source data

Source Data

## Data Availability

The genome sequences (PacBio sequences) of M1_UK_ and M1_global_ reference strains generated in this study have been deposited in the ENA database under the BioProject PRJEB68198. Illumina short reads of all 1815 *emm*1 *S*. *pyogenes* used in this study from invasive disease cases (from the UK, 2013 to 2023) were deposited under the BioProject PRJEB68199. Illumina short reads of *emm*1 non-invasive disease pharyngitis isolates collected in London in 2022 were deposited under the BioProject PRJEB71329. Metadata relating to newly sequenced UK isolates and other genome sequences used in this work are provided in Supplementary Data 3 and in associated source data files. Detailed demographic information is protected due to privacy rules. Genome assemblies and metadata of 2365 M1_UK_ isolates analysed in this study are available as a collection on Pathogen Watch (https://pathogen.watch/collection/6pssdapzoqg5-m1uk-and-intermediates-vieira-et-al-2024). Reference genome MGAS5005 is available at https://www.ncbi.nlm.nih.gov/datasets/gene/GCA_000011765.2. Source data are provided in this paper.
