# Supplementary material for: Rapid expansion and international spread of M1UK in the post-pandemic UK upsurge of Streptococcus pyogenes
Source: Nat Commun. 2024 May 10;15:3916. doi: 10.1038/s41467-024-47929-7 (PMC11087535; doi:10.1038/s41467-024-47929-7)
Supplement: Supplementary file 1 — Supplementary Information [file 41467_2024_47929_MOESM1_ESM.pdf]

## Supplementary information Figures and Tables for

## Rapid expansion and international spread of $M1_{UK}$ in the post-pandemic UK upsurge of *Streptococcus pyogenes*

| | |
|---|---|
| Supplementary Table 1 | Signature SNPs of the three emergent clades during iGAS upsurge in England |
| Supplementary Table 2 | SNP variation observed within different $M1_{UK}$ and $M1_{global}$ groups. |
| Supplementary Table 3 | Clinical source of invasive *emm*1 strains used in this study (2013 to 2023) |
| Supplementary Table 4 | *Emm*1 isolates from lower respiratory tract and pleural samples: association with *emm*1 sub lineages over time. |
| Supplementary Table 5 | Differences in phage content in 1815 *emm1* invasive strains |

| | |
|---|---|
| Supplementary Figure 1 | Phylogenetic tree showing *emm*1 isolates from iGAS in children (< 15 years) intermixed with iGAS in adults (2013 to 2023). |
| Supplementary Figure 2 | Differences between lineages in SNP distribution across 1815 emm1 genomes mapped against MGAS5005 |
| Supplementary Figure 3 | Frequency of change in caseinolytic activity after *in vivo* passage of panel of $M1_{global}$ and $M1_{UK}$ strains |
| Supplementary Figure 4 | Non-synonymous mutations in regulatory genes of *emm*1 strains: distribution within the phylogenetic tree |
| Supplementary Figure 5 | Estimated effective population size (Ne) of $M1_{UK}$ in the UK through time. |

**Supplementary Table 1 – Signature SNPs of three emergent $M1_{UK}$ clades identified during iGAS upsurge**

| | Location | Locus | Gene | Product | Genetic change Synonymous (S) Non-synonymous (NS) | Protein change | Allele in $M1_{UK}$ Clade of interest | Allele in remaining $M1_{UK}$ | Allele in $M1_{global}$ |
|---|---|---|---|---|---|---|---|---|---|
| **$M1_{UK}$ Clade 1** (123 strains) | 359672 | M5005_Spy0353* | x | putative membrane spanning protein | STOP 28C>T | Gln10* | T | C | C |
| | 365403 | M5005_Spy0360 | x | NAD-dependent oxidoreductase | S 444C>T | Asp148Asp | T | C | C |
| **$M1_{UK}$ Clade 2** (166 strains) | 1514811[Ψ] | Intergenic region | | | Intergenic | Intergenic | T | C | C |
| | 1681178[β] | M5005_Spy1718 | *sic1.01* | streptococcal inhibitor of complement | NS 770A>T | Gln257Leu | A | T | T |
| | 711809 | M5005_Spy0709 | *pyrC* | dihydroorotase | NS.509A>C | Asn170Thr | C | A | A |
| | 727058 | M5005_Spy0723 | *hflX* | GTP-binding protein | S.813C>T | Thr271Thr | T | C | C |
| | 47867 | M5005_Spy0029 | *purD* | phosphoribosylamine—glycine ligase | S 840C>T | Ile280Ile | T | C | C |
| | 1122436 | M5005_Spy1146 | | DNA polymerase III, delta subunit | NS.643G>A | Asp215Asn | T | C | C |
| **$M1_{UK}$ Clade 3** (285 strains) | 899440 | M5005_Spy0913 | *xerD*[Ω] | recombinase | NS791C>T | Ala264Val | A | G | G |
| | 1732547 | M5005_Spy1771 | *hutU* | urocanate hydratase | NS .98G>T | Arg33Ile | T | G | G |
| | 1493521 | M5005_Spy1534 | *secA* | protein translocase subunit | NS.1331A>G | Asp444Gly | C | T | T |

SNP annotation made using snippy and MGAS5005 (CP000017.2) as a reference.
[†]MGAS5005 (NC_007297) has a different annotation in this location: Intergenic region upstream of M5005_Spy0353;
[‡]One strain in remaining $M1_{UK}$ group had missing data;
[§]One strain in remaining $M1_{UK}$ group and one strain in the $M1_{global}$ group had a large deletion in *sic*.
[Ω]MGAS5005 (NC_007297) has a different annotation in this location, *xerS*.
[X]no assigned gene name

**Supplementary Table 2 - SNP variation observed within different $M1_{UK}$ and $M1_{global}$ groups.**

| | Groups | No. of isolates analysed | No. of SNPs (mean) | No. of SNPs (median) | Interquartile range | Max no. of SNPs | Min no. of SNPs |
|---|---|---|---|---|---|---|---|
| **$M1_{global}$** | $M1_{global\ 2013-2021}$ | 143 | 32 | 34 | 19.8-50.0 | 79 | 0 |
| | $M1_{global2022-2023}$ | 46 | 45 | 55 | 38.0-51.7 | 73 | 0 |
| | All $M1_{global}$ | 189 | 45 | 34 | 22.0-50.5 | 73 | 0 |
| **$M1_{UK}$** | $M1_{UK2013-2021}$ | 551 | 15 | 16 | 5.9-24.0 | 39 | 0 |
| | $M1_{UK2022-2023}$ | 1001 | 22 | 22 | 17.9-25.4 | 50 | 0 |
| | All $M1_{UK}$ | 1552 | 17 | 17 | 18.2-24.4 | 50 | 0 |
| | $M1_{UK}$ Clade1 | 123 | 2 | 2 | 1.2-3.0 | 10 | 0 |
| | $M1_{UK}$Clade2 | 166 | 3 | 3 | 2.3-3.7 | 13 | 0 |
| | $M1_{UK}$Clade3 | 285 | 4 | 3 | 2.9-4.8 | 11 | 0 |

**Supplementary Table 3 – Clinical source of invasive disease *emm1* strains used in this study (2013 to 2023)**

| | Overall 2013-2023 | | | 2013-2021 | | | 2022-2023 | | |
|---|---|---|---|---|---|---|---|---|---|
| **Specimens from invasive disease** | **$M1_{UK}$** | **$M1_{global}$** | **Inter†** | **$M1_{UK}$** | **$M1_{global}$** | **Inter†** | **$M1_{UK}$** | **$M1_{global}$** | **Inter†** |
| Blood | 1069 | 124 | 36 | 356 | 95 | 16 | 713 | 29 | 20 |
| Lower Respiratory Tract* (pleural samples) | 108 (78) | 10 (5) | 7 (6) | 24 (11) | 4 (2) | 1 (1) | 84 (67) | 6 (3) | 6 (5) |
| Invasive others¶ | 194 | 29 | 12 | 69 | 21 | 6 | 125 | 8 | 6 |
| Swab samples‡ | 181 | 26 | 19 | 102 | 23 | 6 | 79 | 3 | 13 |
| Total | 1552 | 189 | 74 | 551 | 143 | 29 | 1001 | 46 | 45 |

†Inter category includes intermediate strains ($M1_{13SNPs}$, $M1_{23SNPs}$, $M1_{22SNPs}$, $M1_{19SNPs}$) and derivatives of $M1_{UK}$ ($M1_{26SNPs}$)

*Samples from lower respiratory tract include pleural. Subset of isolates from pleural samples shown in parentheses

¶Invasive others include samples from joints (n=22); central nervous system (n=10); viscera (n=13); tissue or fluid (n=137); information not provided (n=39); others (14)

‡Swab samples include skin, wound, vagina, upper respiratory tract

**Supplementary Table 4 – *emm1* isolates from lower respiratory tract samples and pleural samples: association with *emm1* sub lineages over time.**

| **Lineages** | **Proportion of each lineage from lower respiratory tract samples** | | | **Proportion of each lineage from pleural samples*** | | |
|---|---|---|---|---|---|---|
| | Pre-upsurge 2013-2021 | Upsurge 2022/2023 | Overall 2013-2023 | Pre-upsurge 2013-2021 | Upsurge 2022-2023 | Overall 2013-2023 |
| **$M1_{global}$** | 2.7% (4/143) | 13.0% (6/46) | 5.2% (10/189)$^{\Psi}$ | 1.4% (2/143) | 6.5% (3/46) | 2.6% (5/189)$^{\S}$ |
| **$M1_{UK}$** | 4.3% (24/551) | 8.3% (84/1001) | 6.9 % (108/1552) | 2.0% (11/551) | 6.7% (67/1001) | 5.0% (78/1552) |
| **$M1_{UK}$ separated by new clades** | | | | | | |
| **$M1_{UK}$ Clade1** (123 strains) | nil | 7.3% (9/123) | 7.3% (9/123) | nil | 6.5% (8/123) | 6.5% (8/123)$^{\S}$ |
| **$M1_{UK}$ Clade2** (166 strains) | nil | 7.8% (13/166) | 7.8% (13/166) | nil | 6.6% (11/166) | 6.6% (11/166)$^{\S¶}$ |
| **$M1_{UK}$ Clade3** (285 strains) | nil | 10.2% (29/285) | 10.2% (29/285)$^{\Psi}$ | nil | 8.4% (24/285) | 8.4% (24/285)$^{\S¶}$ |
| **$M1_{UK[excluding\ clades\ 1,2,3]}$** (978 strains) | nil | 7.7% (33/427) | 5.8% (57/978)$^{\Psi}$ | nil | 5.6% (24/427) | 3.5% (35/978)$^{¶}$ |

*Isolates from pleural samples are a subset of lower respiratory tract isolates, collected from pleural empyema.

$^{\S}$Difference between new $M1_{UK}$ clades and $M1_{global}$ empyema isolates significant ($M1_{uk}$ clade1 vs $M1_{global}$ p=0.047; $M1_{uk}$ clade2 vs $M1_{global}$ p=0.036; $M1_{uk}$ clade3 vs $M1_{global}$ p=0.005).

$^{¶}$Difference between new $M1_{UK}$ clades and $M1_{UK[excluding\ clades\ 1,2,3]}$ empyema significant ($M1_{uk}$ clade2 vs $M1_{UK[excluding\ clades\ 1,2,3]}$ p=0.032; $M1_{uk}$ clade3 vs $M1_{UK\ [excluding\ clades\ 1,2,3]}$ p<0.001)

$^{\Psi}$Difference between $M1_{uk}$clade3 and $M1_{global}$ significant (p= 0.029); difference between $M1_{uk}$ clade3 and $M1_{UK[excluding\ clades\ 1,2,3]}$ significant (p=0.005).

Statistical comparison between proportions was undertaken using a one-tailed proportion test between pairs of values using either $M1_{global}$ as the comparator or using $M1_{UK[excluding\ clades\ 1,2,3]}$ as the comparator. No adjustment for multiple comparisons was made.

**Supplementary Table 5 – Differences in phage content in 1815 *emm1* invasive strains**

| Phage | Phage change | No. isolates affected $^{\Omega}$ | Proportion of $M1_{global}$ strains | Proportion of $M1_{UK}$ strains | Superantigen change | DNAse change |
|---|---|---|---|---|---|---|
| **Φ5005.1** | Partial deletion | 11 | 2/189 | 9/1552 | *speA* absent | no change |
| **Φ5005.2** | Partial deletion* | 10 | 8/189 | 2/1552 | no change | *spd3* present |
| **Φ5005.2** | Partial deletion* | 55 | 6/189 | 49/1552 | no change | *spd4* present/*spd3* absent |
| **Φ5005.2** | Present recombinant# | 1 | 0/189 | 1/1552 | no change | *spd3* present |
| **Φ5005.3** | Complete absence | $45^{\Delta}$ | 1/189 | 43/1552 | no change | *sda2* absent |
| **ΦSP1380.vir** | Present | 4 | 1/189 | 3/1552 | *speC* and *ssa* present | *spd1* present |
| **Φ370.1** | Present | $172^{\Psi}$ | 30/189 | 138/1552 | *speC* present | *spd1* present |

* Different versions of partially deleted phage.

\# Recombinant version of phi5005.2 phage.

Δ Includes one intermediate strain ($M1_{22SNPs}$) derived from $M1_{23SNPs}$ that also lost Φ5005.3 phage.

Ψ Includes one intermediate strain ($M1_{22SNPs}$, derived from $M1_{23SNPs}$) and three $M1_{26SNPs}$ strains with Φ370.1 phage.

Ω Denominator includes all 1815 invasive isolates, 74 of which were intermediate strains.

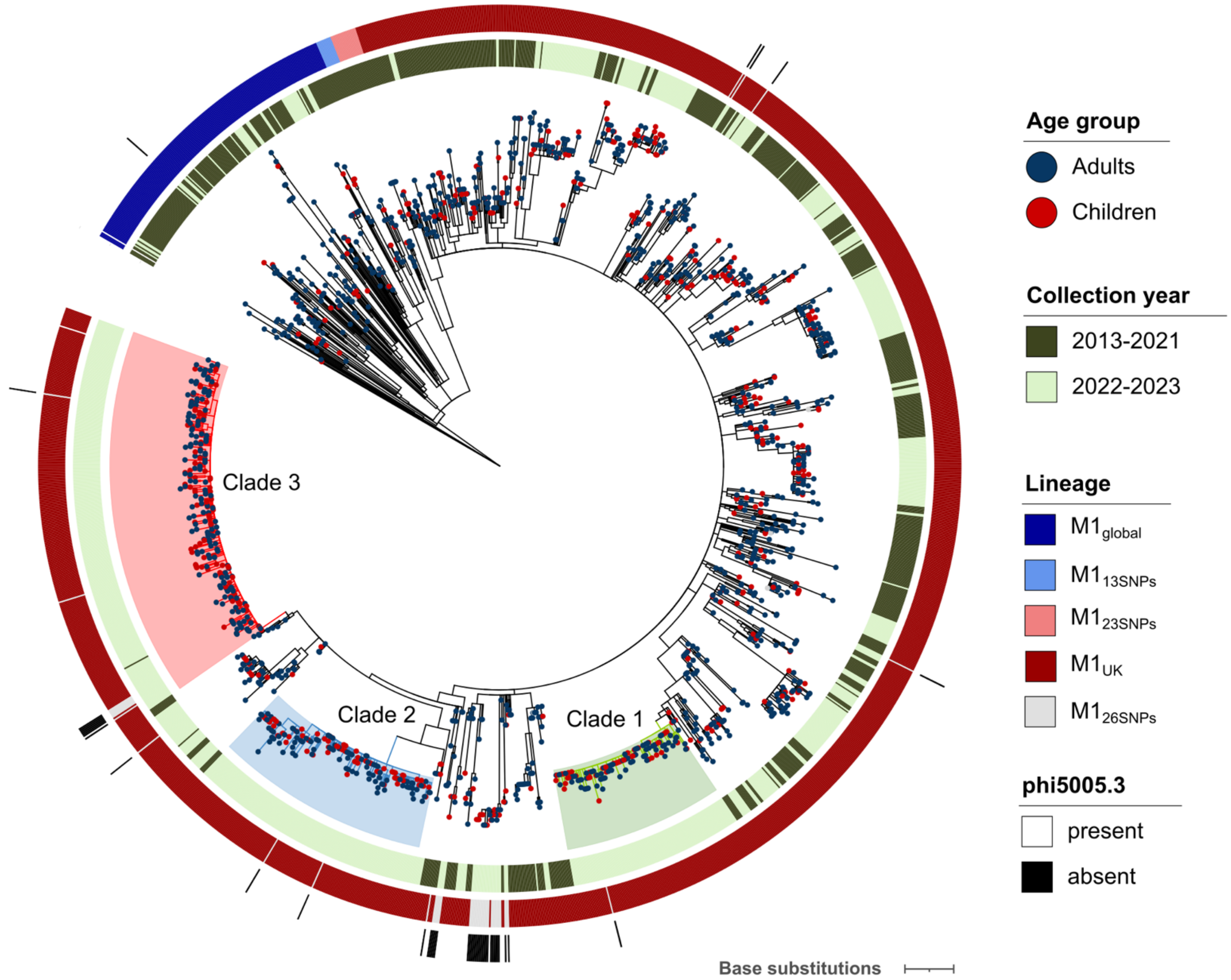


**Supplementary Figure 1 - *emm1* phylogenetic tree showing isolates from iGAS infections in children (< 15 years) intermixed with isolates from iGAS infections in adults (2013 to 2023).** Maximum likelihood phylogenetic tree constructed from 278 core SNPs (excluding recombination regions) extracted after mapping 1815 *emm1* isolated to the MGAS5005 reference genome. The tree was drawn in a circular layout and rooted on outgroup genome NCTC8198. Bars in concentric circles (from inside to outside) are coloured by collection years (pre-upsurge 2013-2021 and upsurge 2022-2023); *emm*1 lineages; and presence-absence of the phi5005.3 phage. The branch tips represent age group (adult or child<15y). Source data are provided as a Source Data file Supplementary Figure 1.

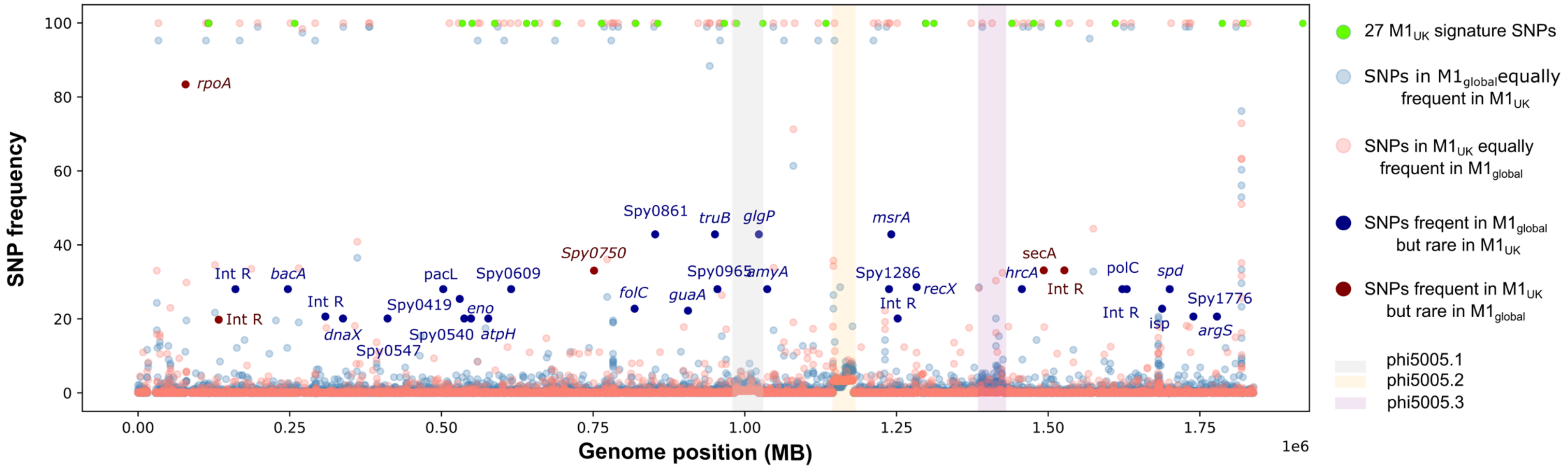


**Supplementary Figure 2 – Differences between lineages in SNP distribution across 1815 *emm1* genomes mapped against MGAS5005** (CP000017.2)**.** The percentage of strains with a specific SNP mutation are indicated. $M1_{UK}$ signature SNPs are coloured in green; SNPs present in both lineages are coloured with a pale colour; SNPs occurring with high frequency (present in >20%) in one of the lineages but rare in the other lineage (frequency < 0.5%) are indicated with darker colours. Int R- indicates Intergenic region. Data processed using python v 3.11.6. script. Resolution of individual SNPs is limited due to Figure scale hence some individual SNPs and position of individual SNPs relative to phages may overlap. Source data are provided as a Source Data file Supplementary Figure 2.

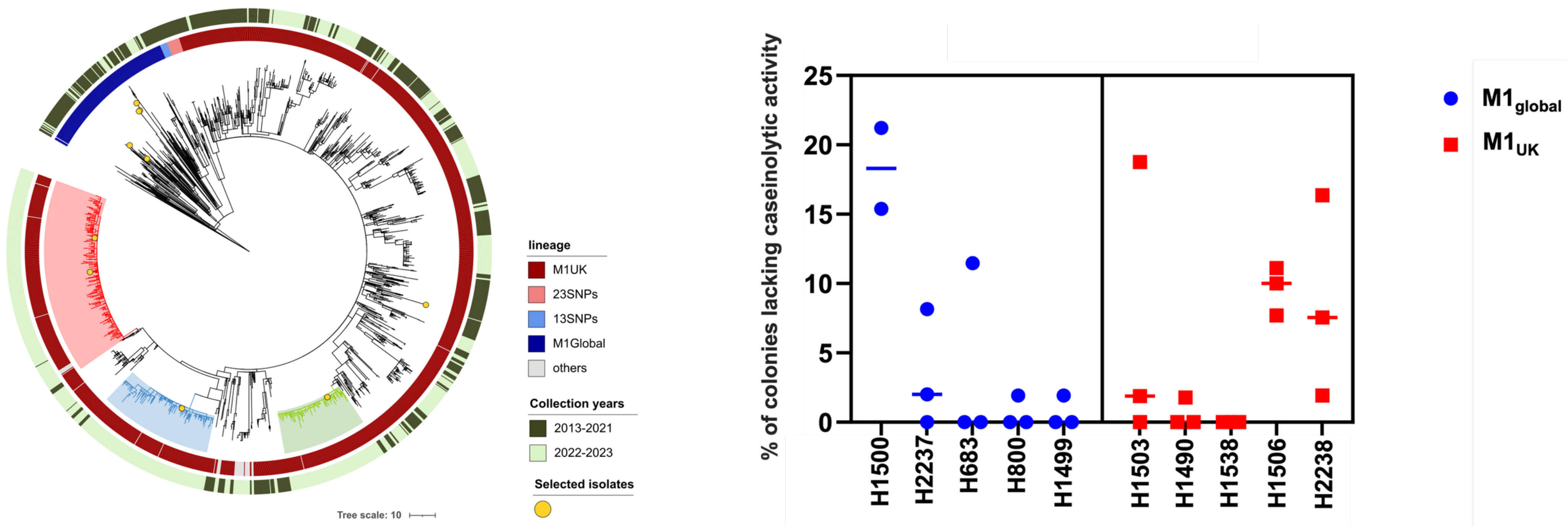


A. B.

**Supplementary Figure 3 Frequency of change in caseinolytic activity after *in vivo* passage of panel of $M1_{global}$ and $M1_{UK}$ strains.**
Ten *S. pyogenes emm*1 strains dating from 2022, that lacked any pre-existing *covS* or *covR* mutation, were selected as representatives of each lineage (5 strains from each of $M1_{global}$ and $M1_{UK}$) as indicated within the *emm*1 phylogenetic tree (A). *S. pyogenes* strains were then used to infect mice intramuscularly, three mice per strain. 50 colonies from each spleen were tested for SpeB activity using a casein digestion assay as a surrogate marker of *covS* mutation. Graph (B) shows percentage of isolates with reduced caseinolytic (SpeB) activity. $M1_{global}$ and $M1_{UK}$ isolates were not significantly different (two-tailed nested t-test, p=0.98). All isolates were previously sequenced and from 2022 *S. pyogenes* non-invasive *emm*1 isolates. $M1_{global}$ strains (H1500, ERS1020045; H2237, ERS1020385; H683, ERS1020523; H800, ERS1020472; H1499, ERS1020620); $M1_{UK}$ (H1503, ERS1020090; H1490, ERS1020174; H1538, ERS1020508; H1506, ERS1020714; H2238, ERS1463088). Source data are provided as a Source Data file Supplementary Figure 3.

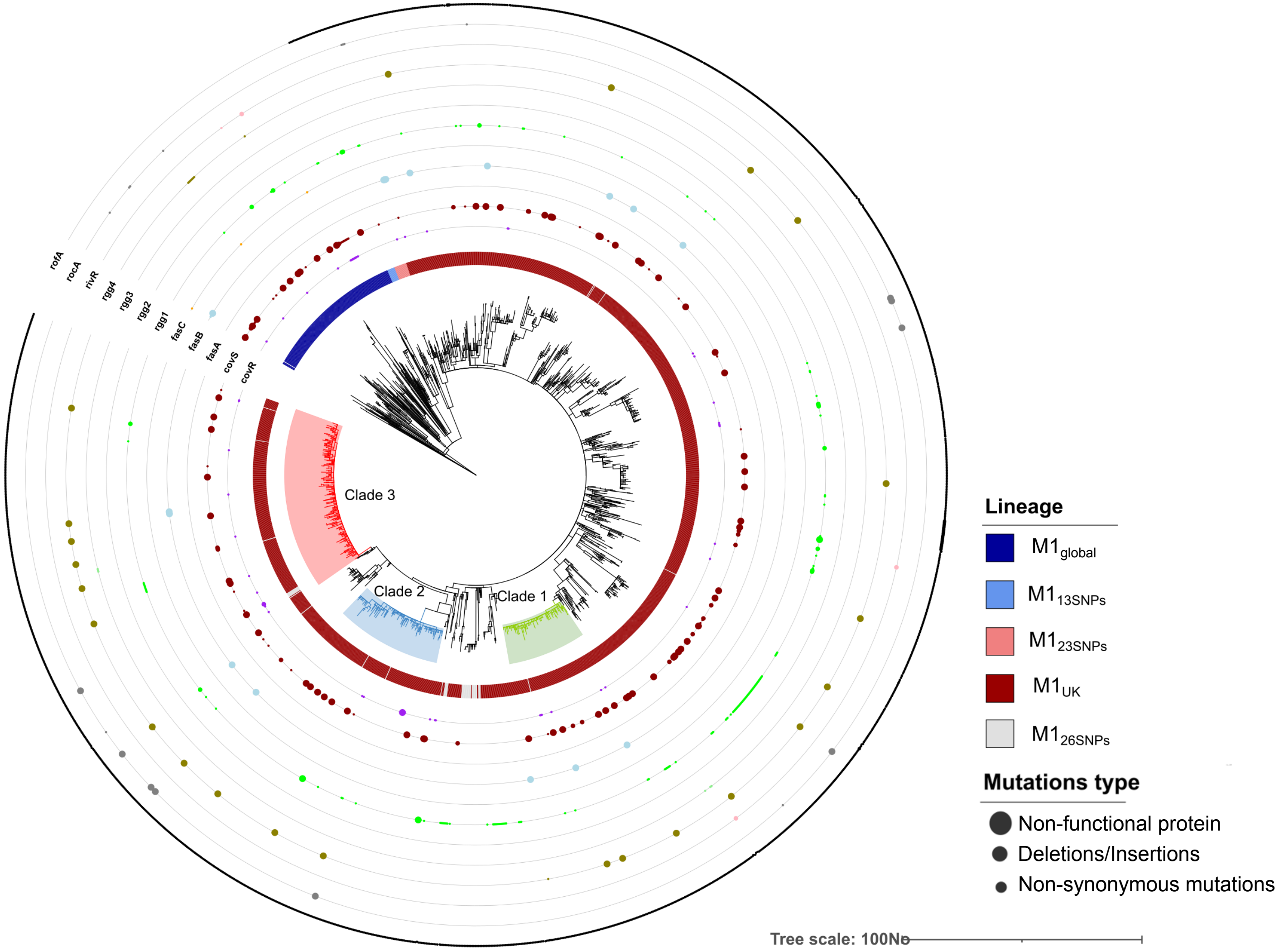


**Supplementary Figure 4 – Non-synonymous mutations in regulatory genes of *emm1* strains: distribution within the phylogenetic tree.** Main phylogenetic tree is central, showing $M1_{global}$, $M1_{UK}$ and associated subclades. Individual regulators are indicated by outer concentric circles. Coloured bubbles indicate a mutation in an individual regulator. Large protein modifications (e.g. truncations) are highlighted with large bubbles; indels and insertions are indicated by medium sized bubbles; non-synonymous mutations are highlighted with small bubbles. All $M1_{UK}$ isolates have 3 missense mutations in RofA (outside ring). Source data are provided as a Source Data file Supplementary Figure 4.

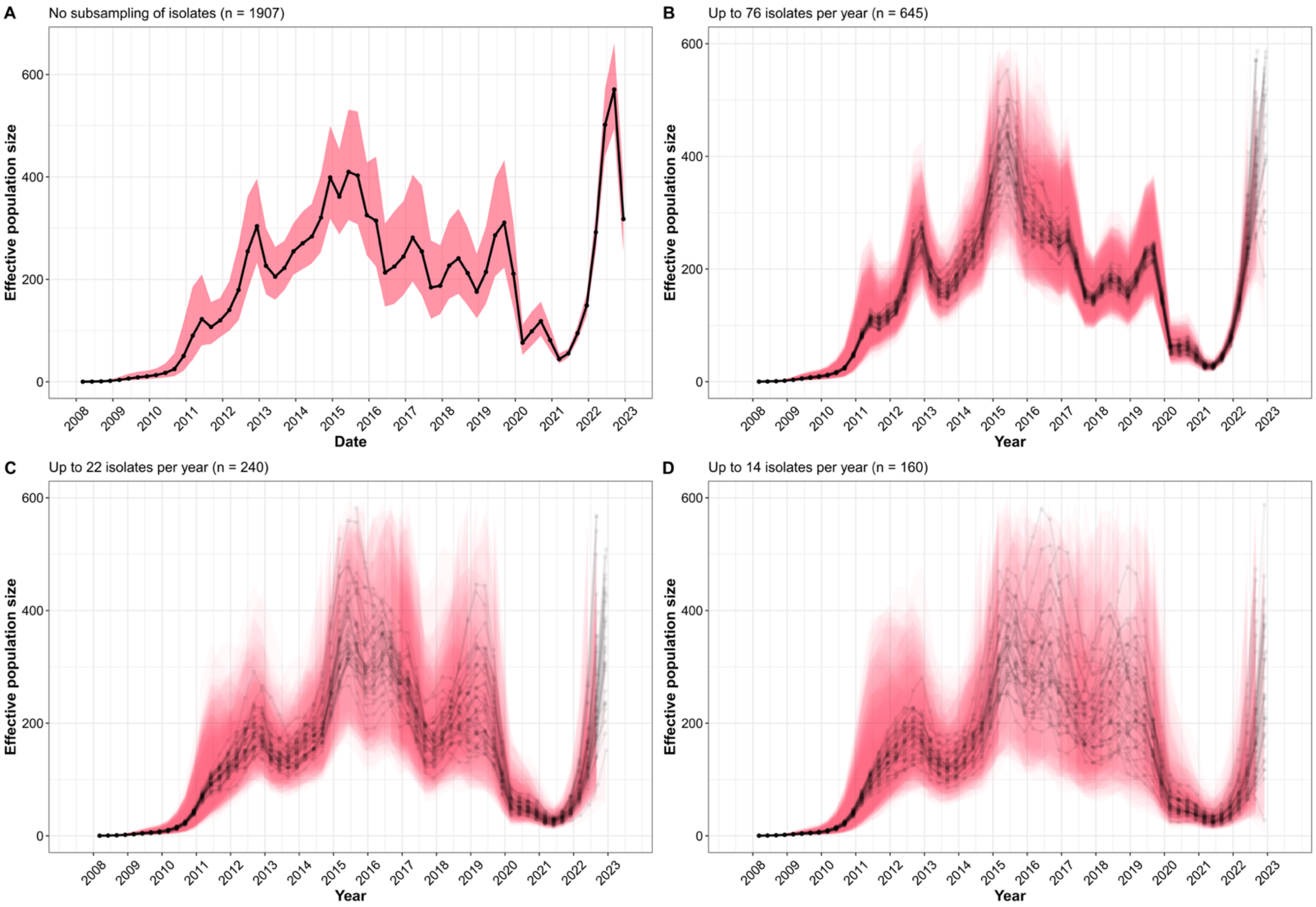


**Supplementary Figure 5. Estimated effective population size ($N_e$) of $M1_{UK}$ in the UK through time.** Panels A-D indicate effect of altering number of isolates considered per year. A) no limit on isolates; B) up to 76 isolates per year; C) up to 22 isolates per year; D) up to 14 isolates per year. The black line and pink shading within each time interval indicate the mean and 95% confidence interval of $N_e$, respectively, estimated from each subtree. Source data are provided as a Source Data file Supplementary Figure 5.
