# Supplementary material for: Rapid expansion and international spread of M1UK in the post-pandemic UK upsurge of Streptococcus pyogenes
Source: Nat Commun. 2024 May 10;15:3916. doi: 10.1038/s41467-024-47929-7 (PMC11087535; doi:10.1038/s41467-024-47929-7)
Supplement: Supplementary file 3 — Description of additional supplementary files [file 41467_2024_47929_MOESM3_ESM.pdf]

Description of Additional Supplementary Files

Supplementary Data 1 : Distribution of indels within 1741 emm1 strains analyzed.

Supplementary Data 2 : Frequency of antibiotic resistance genes and mutations in pbp genes in 1815 emm1 strains analyzed.

Supplementary Data 3 : Metadata and genome accession numbers of emm1 GAS genome sequences used in this study.
