# Supplementary material for: Rapid expansion and international spread of M1UK in the post-pandemic UK upsurge of Streptococcus pyogenes
Source: Nat Commun. 2024 May 10;15:3916. doi: 10.1038/s41467-024-47929-7 (PMC11087535; doi:10.1038/s41467-024-47929-7)
Supplement: Supplementary file 7 — Reporting Summary [file 41467_2024_47929_MOESM7_ESM.pdf]

nature portfolio

Corresponding author(s): Shiranee Sriskandan

Last updated by author(s): Mar 17, 2024

Reporting Summary

Nature Portfolio wishes to improve the reproducibility of the work that we publish. This form provides structure for consistency and transparency in reporting. For further information on Nature Portfolio policies, see our Editorial Policies and the Editorial Policy Checklist.

Statistics

For all statistical analyses, confirm that the following items are present in the figure legend, table legend, main text, or Methods section.

n/a	Confirmed	
☐	☒	The exact sample size (*n*) for each experimental group/condition, given as a discrete number and unit of measurement
☒	☐	A statement on whether measurements were taken from distinct samples or whether the same sample was measured repeatedly
☐	☒	The statistical test(s) used AND whether they are one- or two-sided<br>*Only common tests should be described solely by name; describe more complex techniques in the Methods section.*
☒	☐	A description of all covariates tested
☒	☐	A description of any assumptions or corrections, such as tests of normality and adjustment for multiple comparisons
☐	☒	A full description of the statistical parameters including central tendency (e.g. means) or other basic estimates (e.g. regression coefficient) AND variation (e.g. standard deviation) or associated estimates of uncertainty (e.g. confidence intervals)
☐	☒	For null hypothesis testing, the test statistic (e.g. *F*, *t*, *r*) with confidence intervals, effect sizes, degrees of freedom and *P* value noted<br>*Give P values as exact values whenever suitable.*
☒	☐	For Bayesian analysis, information on the choice of priors and Markov chain Monte Carlo settings
☒	☐	For hierarchical and complex designs, identification of the appropriate level for tests and full reporting of outcomes
☒	☐	Estimates of effect sizes (e.g. Cohen's *d*, Pearson's *r*), indicating how they were calculated

Our web collection on statistics for biologists contains articles on many of the points above.

Software and code

Policy information about availability of computer code

Data collection

Weekly data relating to clinical samples from ISO-8601 week 1 2016 to week 30 2023 reported by English laboratories to yield S. pyogenes were extracted from the UK Health Security Agency (UKHSA) Second Generation Surveillance System (SGSS) on 7 December 2023.
All isolates from invasive disease are submitted to the national reference laboratory and these were emm genotyped. Isolates identified to be emm1 were identified for this study. Genome sequences of all emm1 isolates from invasive infections in England, Wales and N. Ireland that were submitted to the national reference laboratory 2013-2023 by clinical laboratories, and that were sequenced, were used in this study. All

S. pyogenes isolates from throat swabs submitted routinely to the Imperial College NHS Trust (Northwest London Pathology) diagnostic laboratory in 2022 were genotyped . Genome sequences of those that were identified to be emm1 were used in this study.

Two previously sequenced sore throat strains identified to be M1global or M1UK were used for PacBio sequencing as New reference strains .
Data relating to other genome sequences were obtained from publicly available databases using SRA toolkit (https://github.com/ncbi/sra-tools) or from corresponding authors.
FOr most analyses Reference genome MGAS5005 was used (https://www.ncbi.nlm.nih.gov/datasets/gene/GCA_000011765.2/)

Data analysis

New reference strains were long read sequenced using PacBio. The reads were demultiplexed using Lima v2.2.0 (https://lima.how/) and assembled using Redbean v 2.2542 and Trycycler v0.5.343. The assembly quality was assessed using QUAST v5.0.244 and BUSCO v5.3.045. The annotation was performed using prokka v1.14.6.
All other strains-Illumina NextSeq reads were trimmed with Trimmomatic v0.3947, de-novo assembled with SPAdes v3.15.449 and annotated with prokka v1.14.6. The assembly quality was assessed using QUAST v5.0.244. The SNP calling analysis was performed with snippy v4.6.0 using MGAS5005 or M1UK Pacbio sequenced strain as a reference. The recombinant regions were identified with Gubbins v3.3.0. The maximum-likelihood phylogenetic tree was constructed using RAxML-NG v1.0.1 and visualized with iTOLv6.8.152. A dated phylogenetic tree

was generated from the ML tree using the least-squares dating method implemented in the LSD2 module of IQ-Tree v2.2.2.7. Ancestral geographical locations were inferred from the dated tree using PastML. All analyses are described in detail in Methods.
All analyses are described in detail in Methods

For manuscripts utilizing custom algorithms or software that are central to the research but not yet described in published literature, software must be made available to editors and reviewers. We strongly encourage code deposition in a community repository (e.g. GitHub). See the Nature Portfolio guidelines for submitting code & software for further information.

Data

Policy information about availability of data

All manuscripts must include a data availability statement. This statement should provide the following information, where applicable:

- Accession codes, unique identifiers, or web links for publicly available datasets
- A description of any restrictions on data availability
- For clinical datasets or third party data, please ensure that the statement adheres to our policy

The genome sequences (PacBio sequences) of M1UK and M1global generated in this study have been deposited in the ENA database under the BioProject PRJEB68198 (https://www.ebi.ac.uk/ena/browser/view/PRJEB68198). Illumina short reads of all 1815 emm1 S. pyogenes used in this study from invasive disease cases (from the UK, 2013 to 2023) were deposited under the BioProject PRJEB68199 (https://www.ebi.ac.uk/ena/browser/view/PRJEB68199). Illumina short reads of emm1 non-invasive disease pharyngitis isolates collected in London in 2022 were deposited under the BioProject PRJEB71329 (https://www.ebi.ac.uk/ena/browser/view/PRJEB71329). Metadata relating to newly-sequenced UK isolates and other genome sequences used in this work are provided in Supplementary Data 3 and in associated source data files. Detailed patient-level demographic information are protected due to privacy rules. Genome assemblies and metadata of 2364 M1UK and intermediate isolates analysed in this study are also available as a collection on Pathogen Watch (https://pathogen.watch/collection/6pssdapzoqg5-m1uk-and-intermediates-vieira-et-al-2024). Reference genome MGAS5005 is available at https://www.ncbi.nlm.nih.gov/datasets/gene/GCA_000011765.2/
Source data are provided with this paper for Figures 1-5 and for Supplementary figures 1-5

Research involving human participants, their data, or biological material

Policy information about studies with human participants or human data. See also policy information about sex, gender (identity/presentation), and sexual orientation and race, ethnicity and racism.

Reporting on sex and gender	Analysis by sex or gender was not undertaken
Reporting on race, ethnicity, or other socially relevant groupings	Analysis by race or ethnicity was not undertaken
Population characteristics	The age range of the population with invasive group A streptococcal infection was 0-85 years.<br>As invasive group A strep is a rare condition, to protect patient identification and to simply analysis, patients were considered in just two age ranges (children were 15 years and under; adults were 16 years and older).<br>Bacterial isolates from patients with clinician-reported invasive group A streptococcal disease were categorised by sample source. As the study was observational and focused on bacterial isolates not patient outcomes, characteristics such as treatment group, previous and current diagnosis (beyond invasive group A streptococcus) were not collected.<br>All characteristics of the population from whom bacterial isolates were obtained were determined solely by incidence of disease; no selection was applied beyond requirement for a bacterial isolate to be identified.
Recruitment	There was no participant recruitment. All relevant bacterial isolates cultured from samples that were submitted routinely to a laboratory during the time frame studied were included
Ethics oversight	UK Health Security Agency surveillance of infections for health protection purposes is approved under Regulation 3 of The Health Service (Control of Patient Information) Regulations 2020 and under Section 251 of the NHS Act 2006. The collection and genomic analysis of fully anonymised bacterial isolates previously linked to routine data in west London was approved by a national research ethics committee (West London Research Ethics Committee 06/Q0406/20).

Note that full information on the approval of the study protocol must also be provided in the manuscript.

Field-specific reporting

Please select the one below that is the best fit for your research. If you are not sure, read the appropriate sections before making your selection.

☒ Life sciences ☐ Behavioural & social sciences ☐ Ecological, evolutionary & environmental sciences

For a reference copy of the document with all sections, see nature.com/documents/nr-reporting-summary-flat.pdf

Life sciences study design

All studies must disclose on these points even when the disclosure is negative.

Sample size	The main genomic study included all subjects (samples) that were available in the given time periods- these were unselected.
Data exclusions	The only data excluded were those where whole genome sequences were of insufficient quality to analyse- this is stated in the paper

Replication	This was not a primary experimental study; rather, this was a response to national clinical incident . As such,replication of the study is not feasible except in another country. As reported in the Discussion, other countries have reported similar upsurges in Strep A infections, most reporting M1UK to be involved. However the other studies do not currently provide the number of isolates or quality of sequences that would permit replication or validation of the findings in the current study.
Randomization	This was a largely observational non-interventional non-experimental study and therefore randomisation was not relevant except for the choice of isolates to use for CovS mutation in vivo studies (isolates were selected randomly with the following constraints: throat isolates from 2022, known genome sequence, no prior covR or covS mutation, including at least 5 strains from M1global and at least 5 from M1UK, including at least one strain from clades 1-3).
Blinding	As this was a predominantly observational study, responding to a national incident, blinding was not used except that the metadata relating to samples was only provided after strains were genome sequences and assigned to lineages. For animal experiments, the investigator was blinded as to the lineage of each infecting strain.

Reporting for specific materials, systems and methods

We require information from authors about some types of materials, experimental systems and methods used in many studies. Here, indicate whether each material, system or method listed is relevant to your study. If you are not sure if a list item applies to your research, read the appropriate section before selecting a response.

Materials & experimental systems

n/a	Involved in the study
☒	☐ Antibodies
☒	☐ Eukaryotic cell lines
☒	☐ Palaeontology and archaeology
☐	☒ Animals and other organisms
☐	☒ Clinical data
☒	☐ Dual use research of concern
☒	☐ Plants

Methods

n/a	Involved in the study
☒	☐ ChIP-seq
☒	☐ Flow cytometry
☒	☐ MRI-based neuroimaging

Animals and other research organisms

Policy information about studies involving animals; ARRIVE guidelines recommended for reporting animal research, and Sex and Gender in Research

Laboratory animals	Female BALB/c mice aged 6 weeks bred at a regulated UK facility were used in this work. Mice were maintained in a standard 12h light/12h dark cycle with food and water available ad libitum.
Wild animals	No animals from the wild were used
Reporting on sex	30 female mice were used. The results obtained are expected to be equally relevant to both males and females
Field-collected samples	No field-collected samples were involved
Ethics oversight	All animal experiments were undertaken using protocols approved by the Imperial College Animal Welfare Advisory Board (AWERB) and authorised by a UK Home Office Project Licence.

Note that full information on the approval of the study protocol must also be provided in the manuscript.

Clinical data

Policy information about clinical studies
All manuscripts should comply with the ICMJE guidelines for publication of clinical research and a completed CONSORT checklist must be included with all submissions.

Clinical trial registration	This was not a clinical trial.
Study protocol	The collection of bacterial isolates is part of the surveillance function of UKHSA ; the collection of bacterial isolates is part of an ethically approved research protocol at Imperial College London
Data collection	Bacterial isolates and associated relevant demographic information including sample source are routinely submitted to UKHSA for genotyping as standard clinical practice. Bacterial isolates are also submitted routinely for diagnosis to the laboratories of North west London Pathology . Approval exists to link non identfiable demographic data to the samples
Outcomes	Outcomes were not measured in this study. Bacterial isolates were the subject of study

Plants

Seed stocks	not applicable
Novel plant genotypes	not applicable
Authentication	not applicable
